# Effect of early childhood development interventions implemented by primary care providers commencing in the neonatal period to improve cognitive outcomes in children aged 0–23 months: protocol for a systematic review and meta-analysis

**DOI:** 10.1186/s13643-019-1142-1

**Published:** 2019-08-30

**Authors:** Karen M. Edmond, Natalie A. Strobel, Claire Adams, Dan McAullay

**Affiliations:** Medical School, Division of Paediatrics, University of Western Australia, Perth, Australia

**Keywords:** Primary care, Early childhood development, Neonatal, Cognition

## Abstract

**Background:**

Impacts of early childhood development (ECD) interventions (such as fostering attachment and responsiveness through communication, play and stimulation) are well known. Globally, there is increasing recognition of the importance of the ‘golden’ minutes, hours and days after birth for infant health and development. However, only one systematic review has examined ECD interventions implemented in the neonatal period (0–27 days), and this review only assessed interventions implemented by specialised providers. Primary care providers have many potential contacts with mothers and infants throughout the neonatal period. However, it is unclear how many research studies or programmes have examined the effectiveness of ECD interventions commencing in the neonatal period and which methods were used. To date, there has been no systematic review of the effect of ECD interventions delivered by primary care providers commencing in the neonatal period.

**Methods:**

Our overall aim is to conduct a systematic review of the effect of ECD interventions implemented by primary care providers in the neonatal period. We will assess effects by timing and number (‘dose’) of contacts with primary care providers. Subgroup assessment will include effects in disadvantaged infants such as those born with low birth weight and to mothers with mental health disorders. We will also assess effects in low- and high-income countries and by type of care provider. The primary outcome is cognitive status in children aged 0–23 months as measured using standardised scales. Secondary outcomes include other child neurodevelopment domains (speech, language, fine motor, gross motor, social, emotional, behaviour, executive functioning, adaptive functioning) in children aged 0–23 months. Effects on maternal mental health will also be assessed between 0–23 months postpartum. Databases such as MEDLINE (OVID), PsycINFO (OVID), EMBASE (OVID), CINAHL, Cochrane Library, WHO databases and reference lists of papers will be searched for relevant articles. Only randomised controlled trials will be included. A narrative synthesis for all outcomes will be reported. Meta-analyses will be performed where exposures and outcomes are sufficiently homogeneous. Guidelines for PRISMA-P (Preferred Reporting Items for Systematic Reviews and Meta-Analyses Protocols) will be followed.

**Discussion:**

This review appears to be the first to be conducted in this area. The findings will be an important resource for policymakers, primary care providers and researchers who work with young infants in primary care settings.

**Systematic review registration:**

PROSPERO CRD42019122021

**Electronic supplementary material:**

The online version of this article (10.1186/s13643-019-1142-1) contains supplementary material, which is available to authorized users.

## Background

Over 40% of disadvantaged children under 5 years globally have neurodevelopmental problems resulting in deficits in social, emotional and educational functioning into adulthood [1–3]. Early childhood development (ECD) interventions delivered to children aged under 5 years have been clearly shown to have substantial and sustained impacts on long-term cognition and neurodevelopmental outcomes [4–6] ECD interventions have also been shown to improve maternal mental health outcomes [7, 8]. ECD interventions are defined by the World Health Organization (WHO) as physical, socioemotional, cognitive and motor development interventions implemented between birth and 8 years of age [9].

The importance of the family and social environment in influencing children’s neurodevelopment is well known [10, 11]. However, the impact of health services, especially primary care, is less well understood. Primary care providers include community health workers, generalist nurses, health visitors, midwives, child health nurses and general practitioners. Primary care providers are the first level of health service provision and provide a range of comprehensive community and clinical services. Recent demographic and health surveys indicate that primary care staff provide over 90% of health care for families during the first 5 years of life [12]. Primary care providers are in a unique position to augment early child developmental outcomes prior to school entry [13, 14]. Primary care staff routinely provide health advice, counselling and the promotion of behaviour change for their clients. Pregnant women, families and caregivers of young children receive anticipatory guidance, health promotion, health education, promotional interviewing and motivational interviewing. They also receive screening, surveillance and ‘brief interventions’. Delivery channels include home visiting, mobile health teams, clinic visits, group programmes, telehealth, postnatal care and child health checks [13, 14]. However, many primary care providers lack skills and confidence in developmental care for young children and few receive appropriate training, education and tools.

These observations led to the development of the WHO/UNICEF ‘Care for Child Development’ package (CCD). CCD is focused on improving the skills of health care providers in counselling families about responsive caregiving and stimulation for their children [15, 16]. There has been one large robust evaluation of the effect of CCD delivered by primary care providers. In Pakistan, a large randomised controlled trial (RCT) of CCD delivered by female primary care health workers to families of children aged less than 24 months followed up 1302 children to 4 years of age [17, 18]. Children who received responsive stimulation had significantly higher cognition (effect size [Cohen’s *d*] 0.1 for IQ, mean difference from control 1.2, 95%CI 0.3–2.7), executive functioning (0.3 [0.18, 0.07–0.29]) and prosocial behaviours (0.2 [0.08, 0.03–0.13]) at 4 years than children who did not receive responsive stimulation. Mother’s responsive caregiving behaviours were also higher in the intervention than the control group (0.3 [1.95, 0.75 to 3.15]) at 4-year follow up. Other primary care provider ECD interventions include video feedback, positive parenting, family partnership working, attachment-based interventions and motivational interviewing [19–23]. Four systematic reviews report the important impacts of ECD care packages delivered by primary care providers to families of children under 5 years on child and adult cognitive outcomes [24–27].

Simple interventions implemented by primary care providers in the neonatal period (0–27 days) such as early initiation of exclusive breastfeeding, skin to skin contact and handwashing have been shown to substantially reduce mortality and morbidity in children under 5 years [4, 28, 29]. Globally, there is increasing recognition of the importance of the ‘golden’ minutes, hours and days after birth for infant health and development [30–32]. Knowledge about the effects of ‘neonatal ECD’ interventions, such as responsive stimulation focused on play and communication, is also increasing [6, 15]. There is also clear evidence that the nervous system undergoes major shifts in myelination during the neonatal period, and there is great plasticity especially within the cortical and limbic systems [33]. There are a growing body of data to show that neonates are not simply reflexive and subcortical but have important communication and social behaviours especially eye contact, visual locking, auditory responses, responsiveness and self-quietening behaviour [33–35]. By the end of the first month of life, neonates respond to language, look at bright contrasts, movement and colour. Healthy neonates regulate their physiological states by self-soothing, habituate to repeated sensory inputs and respond to social speech [36, 37]. However, to our knowledge, there has been only one systematic review of the effect of ECD interventions commencing the neonatal period [36]. This review included 16 RCTs of 851 participants and assessed the impact of two specialised tools (the neonatal behavioural assessment scale (NBAS) and neonatal behavioural observation (NBO) tool). Seven studies involving 304 participants contributed data to one meta-analysis of the impact of these tools on caregiver-infant interaction, and the results suggested a significant, medium-sized difference between intervention and control groups (SMD − 0.53, 95%CI − 0.90 to − 0.17), but no other outcomes were reported in the included studies or the systematic review [36]. There appears to have been no systematic review or meta-analysis of the effect of ECD interventions in the neonatal period delivered by primary care providers.

## Objectives

Our overall aim is to conduct a systematic review of the effect of ECD interventions implemented by primary care providers commencing in the neonatal period. Our primary objective is to assess the effects on cognitive outcomes in children aged 0–23 months. Secondary objectives are to assess effects on (1) other childhood neurodevelopmental domains (speech, language, fine motor, gross motor, social, emotional, behaviour) at 0–23 months and (2) maternal mental health outcomes.

We will also assess effects by (1) type of health care provider (i.e. primary care provider vs other), (2) timing of the neonatal contacts with health care providers (i.e. interventions delivered in the first hour, first day, first week and first month of life), (3) timing of any antenatal interventions, and (4) ‘dose’, i.e. number of contacts with health care providers in the neonatal or antenatal periods.

We also will assess effects in subgroups: (1) premature or low-birth weight infants and (2) other highly disadvantaged infants such as those born to mothers with substance use disorders, mental health disorders, mothers living with domestic violence and mothers subjected to other forms of violence such as armed conflict and refugees and (iii) type of primary care provider (community health worker, nurse, doctor, other).

We will also assess effects in specific country and population level strata such as low-income countries and high-income countries, African countries and Asian countries, fragile and conflict-affected countries, countries with restricted levels of female empowerment, and indigenous populations.

## Methods

### Protocol development

Guidelines for PRISMA-P (Preferred Reporting Items for Systematic Reviews and Meta-Analyses Protocols) will be followed (Additional file 1) [38]. The review protocol is registered on the PROSPERO database (CRD42019122021) which is available at https://www.crd.york.ac.uk/PROSPERO/display_record.php?RecordID=122021.

### Criteria for considering studies for inclusion

#### Types of studies

Individual, cluster and quasi RCTs will be included. We will include published abstracts if there is sufficient information to allow us to assess study eligibility and risk of bias. If sufficient information is not available, the study will await assessment pending the publication of the full trial report or the provision of further information by trial authors. We only included randomised controlled trials because we wanted studies with a robust assessment of efficacy to enable our study to inform both research planning and policy.

#### Participants

Infants aged 0–27 days will be included if they can be accessed by mainstream primary care providers in high-, middle- and low-income countries. Targeted and high-risk groups will include (1) premature or low-birth weight infants and (2) other highly disadvantaged infants such as those born to mothers with substance use disorders, mental health disorders and mothers living with domestic violence; mothers subjected to other forms of violence such as armed conflict and refugees will be included as specific subgroups. Studies focused on mothers or children with specific disease entities such as neonatal encephalopathy, malnutrition and HIV will be included and considered as targeted high-risk groups.

#### Intervention

We have defined ECD interventions according to the WHO definition of ‘physical, socioemotional, cognitive and motor development interventions’ [9]. We will especially focus on responsive stimulation and play and communication interventions because these are considered by WHO and UNICEF to be the most effective in improving neurocognitive functioning in the early years [6, 15]. We will only include interventions commencing in the neonatal period (0–27 days) in the presence (i.e. face to face, not in the waiting room or by telehealth) of a generalist primary care provider (health professional who is trained in clinical care, has a recognised clinical qualification and works at the first level of the health system, e.g. community health workers, generalist nurses, health visitors, midwives, child health nurses, general practitioners and other primary care doctors).

The interventions will usually be motivational or educational in nature and use counselling skills. They may include anticipatory guidance, health promotion, health education, promotional interviewing, motivational interviewing and didactic and participatory teaching.

Delivery channels may include home visiting, mobile health team visits, clinic visits, child health checks and group programmes.

We will specifically exclude interventions that do not require the face to face presence of a primary care provider such as interventions provided in the waiting room (e.g. videos and health promotion pamphlets) and telehealth. However, due to the ongoing importance of telehealth interventions, we will maintain a list of telehealth interventions for future study, we will also report on this ‘list’ when reporting on our systematic review results. We will not exclude interventions that are initiated in the neonatal period and continue to, e.g. 12 or 24 months. However, we will exclude interventions that involve medicinal products or procedures such as nutritional supplementation, vaccinations, resuscitation and drug trials. Data on antenatal interventions will be collected but antenatal care is not a specific inclusion criterion.

#### Control condition

The comparator group will be any interventions that do not involve ECD interventions as defined by WHO [9]. This includes any other care, standard care and no care. We are aware that control groups may vary substantially across studies. Thus, we will describe all control groups as carefully as we describe the intervention groups, and we will examine comparative effectiveness across all control groups.

#### Outcomes

Our primary outcome measure is cognitive outcomes in children aged 0–23 months. All measures of cognitive function that have been previously validated as an appropriate test in this domain such as Bayley Scales of Infant and Toddler Development and the Griffiths Mental Development Scales will be included.

Secondary outcomes will include other domains of child neurodevelopment including speech, language, fine motor, gross motor, social, emotional, behaviour, executive functioning and adaptive functioning in children aged 0–23 months as measured using standardised scales. Maternal mental health measured using standardised scales up to 24 months postpartum will also be included.

### Search methods for identification of studies

#### Search strategy

Databases will include the Cochrane Central Register of Controlled Trials (CENTRAL) (The Cochrane Library), MEDLINE (Ovid), EMBASE (Ovid) and the Cochrane Database of Systematic Reviews, Health Technology Assessment (HTA) Database and Database of Abstracts of Reviews of Effects (DARE). An example of the MEDLINE search strategy is in Additional file 2. We will also search clinical trial registries such as Clinical-Trials.gov (http://clinicaltrials.gov/), International Standard Randomised Controlled Trial Number (ISRCTN) (http://www.controlled-trials.com), the WHO International Clinical Trials Registry Platform (http://who.int/ictrp/en/) and the UK Clinical Research Network Study Portfolio (https://www.ukctg.nihr.ac.uk/). The search period will not be restricted and will be in all languages. Translation assistance will be sought.

#### Searching other sources

We will hand-search reference lists from relevant articles to identify further studies. We will contact authors of included studies to determine whether there are any additional studies published, ongoing or unpublished that may be relevant. Although systematic reviews will not be included, we will also search through their reference lists to identify any potentially relevant primary studies.

### Study selection

All titles and abstracts retrieved through the search strategy will be reviewed independently by two authors to identify studies that meet the inclusion criteria. Inclusion criteria at the title and abstract level will be limited to any primary study reporting on interventions that commence in the neonatal period (i.e. interventions that are initiated in the neonatal period but continue to, e.g. 12 months or 24 months will be included). Exclusion criteria at this stage will also be review articles, qualitative and opinion articles and if the study clearly does not include primary care providers and outcomes in children aged under 24 months or their mothers.

Once articles have been identified, full-text articles will be retrieved and independently assessed by two independent review authors. We will specifically look for interventions that commence in the neonatal period (i.e. interventions that are initiated in the neonatal period but continue to, e.g. 12 months or 24 months will be included) in the full-text articles and will exclude all studies that are not restricted to the neonatal period. We will also assess articles for the exclusion criteria as listed above including medicinal interventions such as resuscitation, vaccination and nutritional supplementation. If there is any disagreement, a third study team member will be asked to review the article. For each paper that requires clarification or missing data (including abstract-only publications), we will email the corresponding author. If there is no response, we will email each author one further time. We will document and report on the response rate in our paper, i.e. we will document if the corresponding authors do not reply to our emails. Endnote X7 will be used throughout the process.

### Data extraction and management

Data will be managed using ‘Covidence’ software https://www.covidence.org/home. Data from all included studies will be collected by two independent reviewers using a standardised pretested data collection form. Data collected from each study will include the following:

*Study-level information*. Identification number, authors, journal of publication and citation, year of publication, years of data collection and funding source

*Study design*. Individual, cluster or quasi RCTs

*Country-level information*. Country in which the study was conducted, income level of country (high, middle and low), region (Africa, Asia, Europe, Americas, Oceania), fragile and conflict-affected countries (using World Bank criteria [39]) and indigenous populations

*Participant-level information and subgroups*. Type of participant (mother, infant, family, service provider), number, mean age, age range, gender, target groups, underlying conditions or illnesses of infant (e.g. low birth weight, prematurity, birth asphyxia) and mother (e.g. medical illnesses, substance use disorders, mental health disorders, mothers living with domestic violence, mothers subjected to other forms of violence such as such as armed conflict and refugees), ethnicity, socio-economic status using ‘Progress-plus’ Cochrane criteria [40], and family dynamics (e.g. single parent, other dynamics)

*Health system-level information*. Health facility density (number of primary care facilities per population), distance or time to health facilities

*Intervention group*. Type (WHO/UNICEF CCD, other responsive or cognitive stimulation, other), mechanism (anticipatory guidance, health promotion, health education, counselling, promotional interviewing, motivational interviewing, screening, surveillance, family partnership working), health care provider (community health worker, Indigenous health worker, generalist nurse, health visitor, midwife, child health nurse, general practitioner, other primary care doctor, hospital staff, other), delivery channel (home visiting, mobile health team, clinic visits, group programmes, child health checks), neonatal timing (i.e. interventions delivered in first hour, first day, first week and first month of life) antenatal timing (i.e. first, second, third trimester), dose (i.e. number of contacts with primary care providers in the neonatal or antenatal periods)

*Comparator group*. Full details of other care or standard care

*Outcomes*. Primary: Cognitive function in children aged 0–23 months using scales that have been previously validated as an appropriate test in this domain such as Bayley Scales of Infant and Toddler Development and the Griffiths Mental Development Scales. Secondary: Other domains of child neurodevelopment including speech, language, fine motor, gross motor, social, emotional, behaviour, executive functioning or adaptive functioning in children aged 0–23 months and maternal mental health up to 24 months postpartum. We will also collect age at measurement outcome, participant numbers in each group (numerators and denominators), and crude and adjusted effect sizes reported (e.g. relative risks, mean differences and their 95% confidence intervals [95%CI]).

### Measures of treatment effect

Relative risks and 95%CI will be collected for dichotomous data including the proportion of children reported as having cognitive and neurodevelopmental outcomes above or below particular cut points. We will also collect data on mean differences (MD) for continuous outcomes and standardised mean differences (SMD) if different scales are used together with their 95%CI.

### Dealing with missing data

Where data are missing, we will consider if there are justified methods for imputation. For example, if studies report the mean, without standard deviations (SDs), we will impute the mean SDs from available trials as recommended in the Cochrane Handbook for Systematic Reviews of Interventions [41] and will conduct analysis on an intention-to-treat basis. Otherwise, we will analyse data as reported and we will report any assumptions. We will also investigate, through sensitivity analyses, the effects of any imputed data on pooled effect estimates. We will report on the levels of loss to follow-up and assess this as a source of potential bias.

### Risk of bias assessment

The Cochrane [41] risk of bias assessment tool will be used by two review authors to assess the risk of bias in eligible RCTs. The studies will be categorised as either low, high or unclear risk of bias with explanation for each domain. Risk of bias domains include random sequence generation (selection bias), allocation concealment (selection bias), blinding of participants and personnel (performance bias), blinding of outcome assessment (detection bias), incomplete outcome data (attrition bias), selective reporting (reporting bias) and other bias. Authors will be contacted to provide additional information if data for assessment of the risk of bias from eligible studies are inadequate. All studies will be included regardless of their risk of bias, and risk of bias will be presented using standard methods [41]. Meta-biases including publication bias and selective reporting within studies will also be assessed using standard methods including Funnel plots [41].

In case of disagreement, a third author will be involved in the assessment and resolution. We will not exclude studies based on quality assessments. The information will be used in the analysis and report of the review findings.

### Unit of analysis

All RCTs will be eligible for inclusion. For cluster RCTs, we will first determine if the authors have appropriately controlled for effects of clustering in the study. If there is doubt, the authors will be contacted for clarification. If the error has not been corrected and the data are available, we will derive an estimate of the intracluster correlation coefficient (ICC) from the study using standard methods [41]. If the data are not available, we will determine the ICC using a similar trial or from a study with a similar population. We will report whether an ICC has been used and conduct a sensitivity analysis to determine the effect of using an ICC. If the clustering has been accounted for, we will determine whether this has been adequately completed. If we are unable to adjust for incorrect statistical methods used by the cluster trials and cannot estimate the ICC with any degree of confidence, we will exclude the trial.

### Subgroup and sensitivity analyses

Prespecified subgroups are premature or low-birth weight infants, infants born to mothers with disadvantage (e.g. substance use disorders, mental health disorders, mothers living with domestic violence and mothers subjected to other forms of violence such as armed conflict and refugees) and type of health care provider (community health worker, Indigenous health worker, generalist nurse, health visitor, midwife, child health nurse, general practitioner, other primary care doctor, hospital staff, other).

We will also assess data by prespecified strata: income level of country (high, middle and low) (gross national income, GNI), region (Africa, Asia, Europe, Americas, Oceania), fragile and conflict-affected countries, countries with restricted levels of female empowerment and indigenous populations. We will also perform separate sensitivity analyses for studies with a low, moderate and high risk of bias.

### Data synthesis and analysis

Combined and subgroup data will first be reported in simple descriptive narrative tables. We will pool relative risks and 95% confidence intervals (95%CI) for all outcomes with two or more included studies. Heterogeneity of effects will be assessed visually using forest plots of relative risks, quantified by the *I*2 and tested by *Q* statistic tests [42]. *Q* tests with *p* values < 0.05 or *I*2 values > 50% will be considered to represent substantial heterogeneity. We will use random-effects meta-analysis to calculate weighted mean estimates and 95% confidence intervals across studies for primary and secondary outcomes.

We will use random-effects meta-regression to investigate the effect of GNI, region and the other explanatory variables on the primary outcome while taking account of within-study correlations. We have decided a priori that all effect measures should be adjusted for study design, reporting period, proportion of disadvantaged children, type of primary care provider, timing of the intervention and age of the child at outcome reporting in the final model. Crude and adjusted odds ratios and their 95%CI will be calculated. Statistical analyses will be performed using STATA Release 15 statistical software (Stata, College Station, TX, USA).

### Summary of findings table and GRADE

Two review authors will independently assess the certainty of the evidence (high, moderate, low and very low) using the five GRADE (Grading of Recommendations Assessment, Development and Evaluation) considerations (risk of bias, consistency of effect, imprecision, indirectness and publication bias) [43]. We will also complete a GRADE ‘summary of findings’ table to present our major outcomes and levels of ‘certainty’. In the assessment of our results, we will consider the implementation of the study designs and its limitations (risk of bias), statistical tests for heterogeneity and how different the results are within individual studies (inconsistency), how wide our confidence intervals are within our analyses (imprecision), differences within our populations and interventions (indirectness) and assessment of our funnel plot and the number of studies included in our analysis (publication bias). We will downgrade the certainty of evidence for each outcome if these considerations have not been considered appropriately or as outlined by the GRADE guidelines.

## Discussion

Frequently cited benefit-cost ratios from the USA suggest that for every dollar invested in ECD services, there will be at least a $2 return to society [44, 45]. The economic return has been estimated at between 15 and 17% for every dollar taking into account crime, education and welfare savings, and increased taxes due to higher earnings [44, 45]. Interventions implemented in the neonatal period are likely to be even more cost-effective because primary care providers have many potential contacts with mothers and infants throughout the first month of life ranging from birth attendance to postnatal care and early immunisation services [12, 46]. Primary care coverage is high in the early days after birth, even in low-income countries and disadvantaged populations, but then ‘drops out’ with estimates ranging from 80–100% coverage in the early neonatal period to 10–20% by the third month of life [12].

Despite these data, there is little evidence about the effectiveness of current primary care service models in improving quality of neurodevelopmental care in the neonatal period from 0–27 days of life. There has been little opportunity to rigorously test new primary care service models that could improve newborn health outcomes, especially the most disadvantaged children. In this complex population health environment, many research, academic and funding organisations have lost sight of the importance of developing simple tools for upskilling of primary care providers in the earliest months of life. Neonatal care is especially medicalised. Information about ECD interventions delivered by nurses, midwives, community health workers and front-line ‘on the ground’ workers, and simple interventions and support is particularly needed.

The findings of our review will be an important resource for policymakers, primary care providers and researchers in a variety of different settings. Our review will provide data about which studies, if any, have assessed the effects of ECD implemented by primary care providers commencing in the neonatal period. We will provide up to date information of impact on childhood cognitive outcomes in children aged 0–23 months, any longer-term cognitive effects and effects on maternal mental health outcomes. We will also assess effects by timing of postnatal and antenatal contact and dose response. Our study will assist in understanding the impacts of neonatal ECD interventions on premature and low-birth weight infants and other disadvantaged populations.

Our next steps will include the development of clinical trials to fill gaps in knowledge about the effectiveness and cost-effectiveness of neonatal ECD in high-, middle- and low-income settings and to understand the resources required for implementation at scale.

## Additional files

Additional file 1: Search strategy. (DOCX 15 kb)

Additional file 2: PRISMA checklist. (DOCX 22 kb)

## Data Availability

Not applicable

## Abbreviations

CCD: Care for child development
DARE: Database of Abstracts of Reviews of Effects
ECD: Early childhood development
GRADE: Grading of Recommendations Assessment, Development and Evaluation
HTA: Health Technology Assessment Database
ICC: Intracluster correlation coefficient
ISRCTN: International Standard Randomised Controlled Trial Number
NBAS: Neonatal behavioural assessment scale
NBO: Neonatal behavioural observation tool
PRISMA-P: Preferred Reporting Items for Systematic Reviews and Meta-Analyses Protocols
RCT: Randomised controlled trial
UNICEF: United Nations Children’s Fund
WHO: World Health Organization
