# Supplementary material for: Effect of early childhood development interventions implemented by primary care providers commencing in the neonatal period to improve cognitive outcomes in children aged 0–23 months: protocol for a systematic review and meta-analysis
Source: Syst Rev. 2019 Aug 30;8:224. doi: 10.1186/s13643-019-1142-1 (PMC6716939; doi:10.1186/s13643-019-1142-1)
Supplement: Supplementary file 1 — Search strategy. (DOCX 15 kb) [file 13643_2019_1142_MOESM1_ESM.docx]

**WEBAPPENDIX 1. SEARCH STRATEGY**

| [# ▲](http://ovidsp.tx.ovid.com.ezproxy.library.uwa.edu.au/sp-3.32.1b/ovidweb.cgi?&S=EKKNFPLBPJDDMLGENCDKKBOBGLBBAA00&Sort+Sets=descending) | **Searches** | **Results** |
| --- | --- | --- |
| 1 | (health adj2 (improv$ or promotion)).ti,ab. | 73862 |
| 2 | exp health promotion/ | 70915 |
| 3 | anticipatory guidance.mp. | 1190 |
| 4 | motivational interviewing.mp. or exp Motivational Interviewing/ | 3843 |
| 5 | counsel$.mp. | 121300 |
| 6 | care for child development.mp. | 24 |
| 7 | responsive stimulation.mp. | 63 |
| 8 | cognitive stimulation.mp. | 670 |
| 9 | exp Child Development/ | 56037 |
| 10 | early intervention/ | 2675 |
| 11 | 1 or 2 or 3 or 4 or 5 or 6 or 7 or 8 or 9 or 10 | 305833 |
| 12 | randomized controlled trial.pt. | 475004 |
| 13 | controlled clinical trial.pt. | 92881 |
| 14 | randomi#ed.ab. | 517387 |
| 15 | randomly.ab. | 304108 |
| 16 | trial.ab. | 451497 |
| 17 | clinical trials as topic.sh. | 185783 |
| 18 | 12 or 13 or 14 or 15 or 16 or 17 | 1318514 |
| 19 | exp animals/ not humans.sh. | 4538799 |
| 20 | 18 not 19 | 1211914 |
| 21 | infant/ | 752167 |
| 22 | newborn/ | 574321 |
| 23 | (newborn or neonate or neonatal or infan* or neonat*).tw. | 642802 |
| 24 | 21 or 22 or 23 | 1339926 |
| 25 | 11 and 20 and 24 | 4264 |
